# Fascioliasis Presenting With Migrating Liver Masses

**DOI:** 10.4269/ajtmh.21-0539

**Published:** 2021-07-12

**Authors:** Hiroaki Nishioka, Shohei Fujita, Shigeo Hara

**Affiliations:** ^1^ Department of General Internal Medicine, Kobe City Medical Center General Hospital, Kobe, Hyogo, Japan;; ^2^ Department of Pathology, Kobe City Medical Center General Hospital, Kobe, Hyogo, Japan

A 70-year-old Japanese woman visited a local hospital for intermittent fever and decreased appetite. Her blood test results showed an increased eosinophil count. Contrast-enhanced computed tomography (CT) revealed low-density areas in the liver (Figure 1A). A malignant disease, particularly lymphoma, was suspected, but the diagnosis was not confirmed. Her symptoms persisted and she was referred to our institution. She lived near a ranch. She did not have pets, travel abroad, or eat raw crab, raw fish, or raw meat. She ate vegetables that she grew in her kitchen garden. Physical examination results were unremarkable for any respiratory, cardiovascular, and gastrointestinal findings. Laboratory findings showed a white blood cell count of 14,000/µL (eosinophil count of 6,020/µL), hemoglobin level of 11.5 g/dL, platelet count of 35.2 × 10^4^/µL, aspartate aminotransferase level of 129 IU/L, alanine aminotransferase level of 225 IU/L, and C-reactive protein level of 1.2 mg/dL. Contrast-enhanced CT revealed the disappearance of previous lesions (Figure 1B) and the appearance of new multilocular low-density areas (Figure 1C). Liver biopsy results and histological findings revealed granuloma with eosinophil infiltration and Charcot-Leyden crystals (Figure 2), which suggested a parasitic infection. Parasite eggs and adult parasites were not detected in the stool, bile duct, or liver specimen. However, serological testing for parasites using a microplate ELISA revealed anti-*Fasciola hepatica* antibodies in the serum. An ELISA using recombinant *Fasciola* cathepsin L1 also yielded positive results. Therefore, fascioliasis was diagnosed. She received triclabendazole (500 mg) for 2 days. Subsequently, her fever and eosinophilia subsided, and the low-density areas in the liver diminished in size.

**Figure 1.**
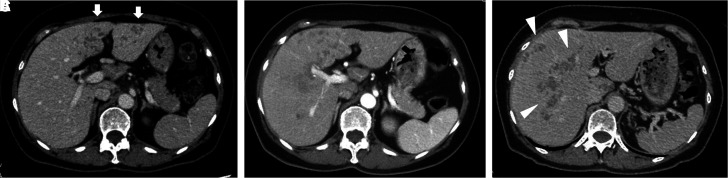
Contrast-enhanced computed tomography of the abdomen. (**A**) Low-density areas are visible in the liver (white arrows). (**B**) The previous lesions disappeared. (**C**) New multilocular low-density areas are seen (white arrows).

**Figure 2.**
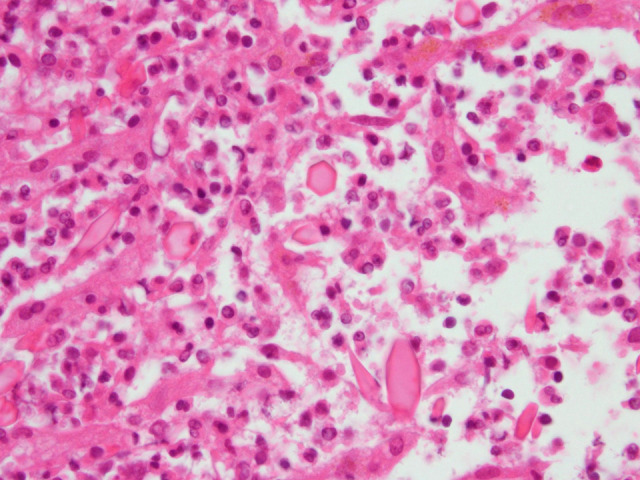
Liver biopsy specimen. Hematoxylin and eosin slide image showing scattered Charcot-Leyden crystals with numerous eosinophils. This figure appears in color at www.ajtmh.org.

Fascioliasis is an infection caused by the liver trematode *Fasciola hepatica*. Most cases are caused by the consumption of contaminated water plants.^1^ Fascioliasis is rare in developed countries; therefore, the diagnosis can be difficult and delayed.^2^ In Japan, some cases have been observed in individuals who live or work at small-scale cattle ranches and their neighbors,^3^ like our patient. Although we could not determine the infection route in this case, we hypothesized that the vegetables grown in her kitchen garden might have been contaminated. The CT findings of fascioliasis include subcapsular low-attenuation areas with an undefined border in the liver.^4^ In the present case, the CT image showed migration of the subcapsular low-density areas in the liver that reflected the parasite movement. In summary, parasitic infections should be included in the differential diagnosis when evaluating patients with liver masses and eosinophilia. This case suggested that migrating low-density areas in the liver observed using CT aided in the diagnosis of fascioliasis.

## References

[1] Graham CS Brodie SB Weller PF , 2001. Imported *Fasciola hepatica* infection in the United States and treatment with triclabendazole. Clin Infect Dis 33: 1–5. 11389487 10.1086/320870

[2] Marcos LA Terashima A Gotuzzo E , 2008. Update on hepatobiliary flukes: fascioliasis, opisthorchiasis and clonorchiasis. Curr Opin Infect Dis 21: 523–530. 18725803 10.1097/QCO.0b013e32830f9818

[3] Ishii Y Nakamura-Uchiyama F Nawa Y , 2002. A praziquantel-ineffective fascioliasis case successfully treated with triclabendazole. Parasitol Int 51: 205–209. 12113760 10.1016/s1383-5769(02)00004-1

[4] Dusak A Onur MR Cicek M First U Ren T Dogra VS , 2012. Radiological imaging features of fasciola hepatica infection - a pictorial review. J Clin Imaging Sci 2: 2. 22347685 10.4103/2156-7514.92372 PMC3279695

